# The Compression Fracture of Thoracic Spine Due to Methotrexate-Associated Lymphoproliferative Disorder in Rheumatoid Arthritis: A Case Report

**DOI:** 10.7759/cureus.78960

**Published:** 2025-02-13

**Authors:** Hisanori Ikuma, Tomohiko Hirose, Dai Nakamura, Satoko Nakamura, Keisuke Kawasaki

**Affiliations:** 1 Department of Orthopaedic Surgery, Kagawa Prefectural Central Hospital, Kagawa, JPN; 2 Department of Pathology, Kagawa Prefectural Central Hospital, Kagawa, JPN

**Keywords:** compression fracture, methotrexate-associated lymphoproliferative disorder, rheumatoid arthritis, thoracic spine, withdrawal

## Abstract

Patients with rheumatoid arthritis (RA) are at high risk of developing cardiovascular disease, infections and malignancies during their lifetime, with lymphoma being the most common malignancy. These patients may clinically present with a wide spectrum of lymphoid proliferations or lymphomas, which are called lymphoproliferative disorders (LPD). The prediction sites of LPD can be divided into nodal and extranodal lesions, with the majority being extranodal lesions. The sites of extranodal lesions especially in RA patients with LPD receiving methotrexate (MTX) are the pharynx, liver, spleen, lung, soft tissues, adrenal gland, pleura, bone, kidney, small bowel and breasts. Among these, bone lesions are rare, with the frequency of occurrence in the spine being extremely rare. In this report, we describe a 76-year-old woman with RA suffering from the isolated methotrexate-associated lymphoproliferative disorder (MTX-LPD) in the thoracic spine. This patient complained of unprovoked back pain for two weeks before visiting our hospital. The neoplastic change and the vertebral compression fracture were found at T7, and the pathological examination from needle biopsy of the T7 vertebral body revealed the possibility of diffuse large B-cell lymphoma or MTX-LPD, and no evidence of cancer metastasis. This patient showed clinical improvement after MTX withdrawal and thoracic posterior fusion with spinal instrumentation for T7 compression fracture. At 30 months after MTX withdrawal, the tumoral lesion remained obliterated, and a solid union of T7 was observed.

## Introduction

Recent advancements in pharmaceutical technologies have led to the development of various new rheumatoid arthritis (RA) therapies that have significantly improved the quality of life for patients with RA [1]. However, patients with RA are at high risk of developing cardiovascular diseases, infections, and malignancies during their lifetime [2], with lymphoma being the most common malignancy [3]. These patients may present with various lymphoid proliferations or lymphomas, known as lymphoproliferative disorders (LPD). LPD in a patient with RA receiving methotrexate (MTX) was first reported by Shiroky et al. in 1991 [4].

According to the World Health Organization (WHO) Classification of Tumours of Haematopoietic and Lymphoid Tissues, revised 4th edition, LPD is classified as ‘other iatrogenic immunodeficiency-associated LPDs’ [5], because it occurs more frequently in patients receiving immunosuppressive drugs. MTX and tumour necrosis factor inhibitors are listed as related drugs [6]. It has become clear that LPD in patients with RA tends to be found more frequently in patients treated with MTX and biological disease-modifying antirheumatic drugs over a long time [7].

Predilection sites for LPD can be divided into nodal and extranodal lesions, with the majority being extranodal lesions. The sites of extranodal lesions, especially in patients with RA having LPD receiving MTX, include the pharynx, liver, spleen, lung, soft tissues, adrenal gland, pleura, bone, kidney, small bowel, and breast [6]. The intraosseous onset is rare, with an incidence of approximately 3% [8], and their occurrence in the spine is extremely rare. Here, we describe a case of isolated MTX-associated LPD (MTX-LPD) of the thoracic spine.

## Case presentation

A 76-year-old woman (height 142.5 cm, weight 42.15 kg) complained of unprovoked back pain around the thoracic spine for two weeks before visiting our hospital. Her back pain was worsened in standing position or walking. Moreover, she felt a rest pain at night. During this period, she experienced night sweats, low-grade fever (from 37.5˚C to 38.3˚C), and a loss of body weight (3 kg). These are considered as possible B symptoms. B symptoms are commonly associated with lymphoma, including fever above 38˚C, drenching night sweats, and weight loss of more than 10% of body mass over the last six months. B symptoms are signs of a poor prognosis in patients with lymphoma [9]. There was no obvious lymphadenopathy on her body surface. She had been suffering from RA for 15 years and had been taking MTX for 10 years. The latest medication doses were 8 mg/week of MTX and 100 mg/d of celecoxib. Her other medical history included hypertension, uterine cancer (no recurrence after total hysterectomy), and no family history of LPDs. The patient had no history of smoking or alcohol consumption.

Laboratory studies revealed that the neutrophil count had increased to 85.5%, and the lymphocyte count had decreased to 10%. However, the levels of various tumour markers, including sIL-2R and C-reactive protein, were normal. Epstein-Barr virus (EBV) viral capsid antigen antibody immunoglobulin G and EBV nuclear antigen antibody levels were elevated to 10.7% and 2.1%, respectively, but EBV viral capsid antigen antibody immunoglobulin M and EBV early antigen antibody immunoglobulin G levels were not elevated (Table 1). These results suggested that the patient had a history of EBV infection.

**Table 1:** Laboratory findings HbA1c, glycated hemoglobin; sIL-2R, soluble interleukin-2 receptor; EBV, Epstein–Barr virus; EBV-VCA, Epstein–Barr virus-viral capsid antigen; IgG, immunoglobulin G; IgM, immunoglobulin M; EBNA, EBV-nuclear antigen; EA, early antigen; N.A., not applicable.

Investigations	Values	Reference range	Units
White blood cells	6.2	3.3–8.6	x1000/μL
Neutrophils	85.5	41.7–74.1	%
lymphocyte	10	17-58	%
Hemoglobin	13.3	11.6–14.8	g/dL
Platelet counts	16.9	15.8–34.8	x1000/μL
Total protein	7.4	6.6–8.1	g/dL
Albumin	4.5	4.1–5.1	g/dL
Blood-urea-nitrogen	21.5	8–20	mg/dL
Creatinine	0.75	0.46–0.79	g/dL
C-reactive protein	0.05	< 0.14	mg/dL
HbA1c	5.1	4.9–6.0	%
Total bilirubin	0.7	0.4–1.5	mg/dL
Aspartate aminotransferase	23	13–30	U/L
Alanine aminotransferase	22	7–23	U/L
Carcinoembryonic antigen	2.8	0.4–5.2	ng/mL
Carbohydrate antigen 19-9	11	3.2–36.8	U/mL
sIL-2R	255	145–519	U/mL
EBV-VCA IgG	10.7	< 10	Fold
EBV-VCA IgM	0.4	< 1.0	N.A.
EBV-EBNA IgG	2.1	< 1.0	N.A.
EBV-EA IgG	0.3	< 1.0	N.A.

**Figure 1:**
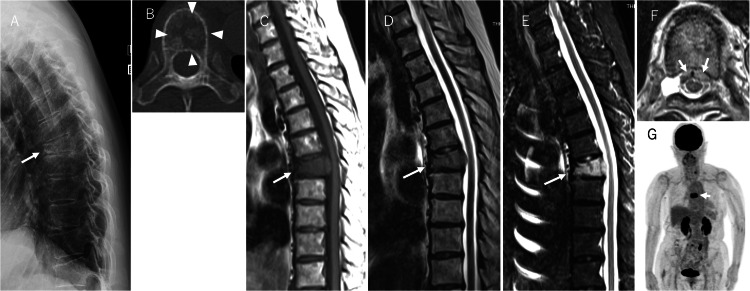
Imaging findings before MTX withdrawal. (A) Lateral view on X-ray at the first visit shows a reduction in the T7 vertebral body height and an increase in thoracic kyphosis (white arrow). (B) Axial view of the T7 vertebral body on CT shows osteolytic changes on the left side of the T7 vertebral body (white triangle). (C) Sagittal view on the T1-weighted MRI shows hypointense changes in the T7 vertebral body (white arrow). (D) Sagittal view on the T2-weighted MRI shows hypointense changes in the T7 vertebral body (white arrow). (E) Sagittal view of T2-weighted short-tau inversion-recovery (STIR) MRI shows hyperintense changes in the T7 vertebral body (white arrow). (F) Axial view of the T7 vertebral body on MRI shows the toxic twin-leaf sign around the posterior wall of T7 (white arrow). (G) Fluorine-18 fluorodeoxyglucose positron emission tomography/computed tomography (18F-FDG PET/CT) shows strong uptake in the T7 vertebral body (white arrow). MTX, methotrexate; CT, computed tomography; MRI, magnetic resonance imaging.

An X-ray scan at the first visit showed a reduction in the T7 vertebral body height and an increase in thoracic kyphosis (Figure 1A). Computed tomography (CT) revealed osteolytic changes on the left side of the T7 vertebral body (Figure 1B). Magnetic resonance imaging (MRI) showed hypointense changes on T1- and T2-weighted images and hyperintense changes on T2 short-tau inversion recovery (STIR) images of the T7 vertebral body (Figure 1C-1E). A toxic twin-leaf sign [7] was found around the posterior wall of the T7 vertebra, indicating neoplastic changes, which initially led to the suspicion of a spinal tumour (Figure 1F). Based on these imaging findings, a compression fracture of the T7 vertebra due to a spinal tumour was suspected. Subsequently, whole-body contrast-enhanced CT was performed to investigate a suspected metastatic tumour; however, there was no evidence of a primary tumour of a vital organ. Fluorine-18 fluorodeoxyglucose positron emission tomography (18F-FDG PET) /CT showed uptake at the T7 vertebra. However, it was difficult to distinguish it from a neoplastic lesion because the patient already had a vertebral compression fracture at the T7 vertebra (Figure 1G). At this point, we suspected the primary tumour or metastatic tumour of the T7 vertebra for the diagnosis of this patient.

**Figure 2:**
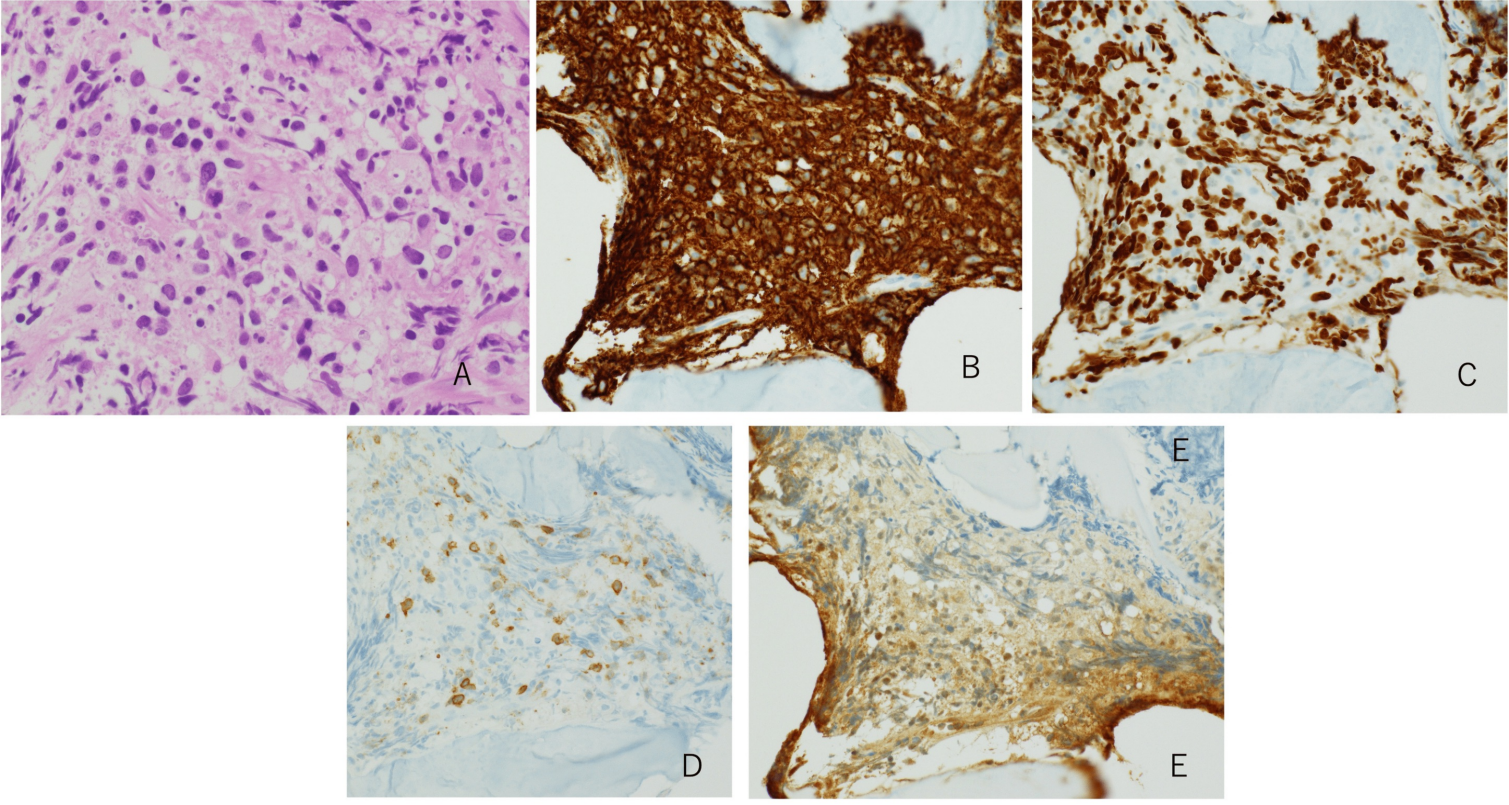
Pathological findings from the needle biopsy under CT scan of the T7 vertebral body. (A) Haematoxylin and eosin stain reveals the proliferation of medium-sized lymphoid cells infiltrating the bone marrow. (B) Lymphoid cells were positive for CD20. (C) Lymphoid cells were also positive for Ki67. (D) Lymphoid cells were negative for CD3. (E) EBER-ISH: Epstein–Barr virus was not detected by in situ hybridisation. Magnification: A, B, C, D, E ×200. EBER-ISH, Epstein–Barr virus-encoded RNA in situ hybridisation; CT, computed tomography.

For these reasons, a needle biopsy of the T7 vertebral body under CT guidance was performed for a definitive diagnosis. The biopsy revealed the proliferation of medium-sized lymphoid cells infiltrating the bone marrow. Immunostaining was negative for CD3 and CD5 but positive for CD20 and Ki67. EBV-encoded RNA was negative on in situ hybridisation. Considering both the pathological findings and the MTX treatment history, the pathologist indicated the possibility of diffuse large B-cell lymphoma (DLBCL) or MTX-LPD, and no evidence of cancer metastasis was found (Figure 2).

**Figure 3:**
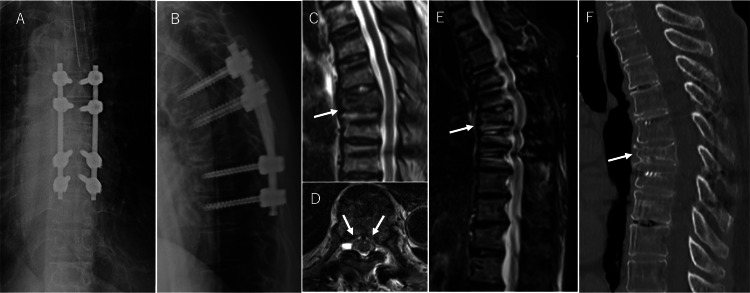
Imaging findings after MTX withdrawal and posterior fusion of the thoracic spine. (A) Anteroposterior (A-P) view on postoperative X-ray shows the thoracic posterior fusion with spinal instrumentation from T5 to T9. (B) Lateral view on postoperative X-ray shows the posterior fusion with spinal instrumentation from T5 to T9. (C) Sagittal view on T2-weighted MRI two weeks after MTX withdrawal shows tumour regression of the T7 vertebra (white arrow). (D) Axial view of the T7 vertebral body on MRI shows that the toxic twin-leaf sign had almost disappeared around the posterior wall of the T7 vertebra (white arrow). (E) Sagittal view on MRI (STIR) 30 months after MTX withdrawal shows that the tumoral lesion remains obliterated (white arrow). (F) Sagittal view on CT scan 30 months after MTX withdrawal shows a solid union of T7 vertebra, bridging to the caudal vertebrae (white arrow). MTX, methotrexate; MRI, magnetic resonance imaging; CT, computed tomography.

The MTX treatment was discontinued after these results were obtained. Posterior spinal fixation for the T7 thoracic compression fracture was performed immediately after the withdrawal of MTX (Figure 3A, 3B). Her severe back pain improved remarkably, and tumour regression of the T7 vertebra was observed on MRI two weeks after MTX withdrawal (Figure 3C, 3D). Thirty months after MTX withdrawal, the tumour remained obliterated, and a solid union of the T7 vertebra was observed (Figure 3E, 3F). Her RA activity was also stable with non-steroidal anti-inflammatory drug administration after MTX withdrawal. Although she did not experience a recurrence of back pain, she was carefully followed up because of the possibility of local or remote recurrence of MTX-LPD.

## Discussion

MTX-LPD is a lymphoproliferative disease that occurs during treatment with the immunosuppressive drug MTX. This pathological condition was first described by Ellman et al. in 1991 as a lymphoma in patients with RA treated with low-dose MTX [10]. The World Health Organization (WHO) Classification of Tumours of Haematopoietic and Lymphoid Tissues, revised 4th edition, classifies MTX-LPD as another iatrogenic subgroup of immune deficiency-associated LPDs [5]. Of these, intraosseous onset is rare, with an incidence of approximately 3% of all cases [8]. Furthermore, reports of thoracic spinal onset are extremely rare. To our knowledge, only three papers discussing thoracic spine onset have been published to date (Table 2) [11-13]. The present case is a case of the thoracic spine origin that had the longest follow-up period of 30 months after MTX withdrawal.

**Table 2:** Previous reports about methotrexate-associated lymphoproliferative diseases (MTX-LPD) in thoracic spine

Author	Year	Country	Age, Sex	Site of lesion	Decisive factor of diagnosis	Anti-EBV capsid antigen- IgG	Treatment	Color of tumor	Follow-up	Recurrence
Kamio et al. [11]	2021	Japan	54 y, F	T10	1. Intraoperative pathological specimen (Classical Hodgkin lymphoma). 2. History of MTX treatment. 3. Tumor remission on CT after MTX withdrawal	High	MTX withdrawal ＋ Posterior fixation	Dark brown	18 months	-
											Hirata et al. [12]	2021	Japan	76 y, M	T6-7	1. History of MTX treatment. 2. Tumor remission on CT after MTX withdrawal	Negative	MTX withdrawal	N.A.	24 months	-
																						Tsukamoto et al. [13]	2022	Japan	69 y, M	T7	1. Intraoperative pathological specimen (Hodgkin's lymphoma-like LPD). 2. History of MTX treatment. 3. Tumor remission on CT and MRI after MTX withdrawal	High	MTX withdrawal ＋ Posterior fixation	Dark brown	12 months	-

Regarding the risk of developing MTX-LPD in patients with RA receiving MTX, those with a history of MTX use for more than 30 months and MTX doses of more than 8 mg/week are believed to be at high risk [10]. In patients with RA, a history of EBV infection is closely associated with lymphoma development. An immunosuppressive state can reactivate EBV in the human body, which may increase the risk of lymphoma development [14]. Regarding the effect of MTX on the regulation of EBV genes, the prompt regression of EBV-positive LPD in patients with RA who are treated with MTX after cessation of the drug suggests that MTX has possible effects on the regulation of EBV gene expression that is relevant to replication of EBV [15]. EBV positivity in patients who have discontinued MTX was significantly higher in spontaneously regressed patients (85.2%) than in non-regressed patients (50%) [16].

This patient had a history of EBV infection, and although the pathology of the needle biopsy was negative for EBV, MTX withdrawal resulted in tumour regression and improvement in her clinical symptoms.

Evaluation of MTX-LPDs is usually performed using MRI. A characteristic finding is that the tumour area shows low signal intensity on both T1-weighted and T2-weighted images and hyperintense changes on STIR images. However, it is difficult to differentiate MTX-LPDs from neoplastic diseases based on imaging findings alone.

Unfortunately, no report has yet provided clear guidelines for the definitive diagnosis of MTX-LPD; however, all reports agree on the importance of a comprehensive evaluation of the clinical course, including a history of cancer, 18F-FDG PET/CT imaging, EBV testing, local histology, and MTX withdrawal. In many reports, tumour regression within 3-8 months after MTX withdrawal was the basis for a definitive diagnosis [17]. In the present case, tumour regression was observed just two weeks after MTX withdrawal and posterior spinal fixation. The posterior spinal fixation should have acted as a strong spinal stabilization for the compression fracture. This stabilization effect might have accelerated the tumour regression.

The most common histological subtype is DLBCL, which accounts for approximately half of all cases, followed by Hodgkin lymphoma, which accounts for 10-20% of cases [18]. The histopathology in the present case resulted in the diagnosis of DLBCL or MTX-LPD.

A minimum follow-up of two years is required after tumour regression, as it has been reported that approximately 30% of patients relapse within 14 months [19]. A relationship between the MTX dosage before discontinuation and the risk of MTX-LPD relapse is still currently unclear. Yamaguchi et al. have recommended the administration of abatacept and tocilizumab for active RA after remission of LPD with reference to the recommendation of the 2015 American College of Rheumatology guidelines [20-21]. For non-responders to MTX withdrawal, conventional chemotherapy for malignant lymphomas, such as R-CHOP (rituximab, cyclophosphamide, doxorubicin, vincristine, and prednisolone), is applicable [22].

In the present case, a tumour lesion was found in the T7 vertebral body; however, subsequent whole-body contrast CT scan and blood tests, including tumour markers, did not provide evidence of neoplastic disease. 18F-FDG PET/CT showed uptake at the T7 vertebra; however, this was not considered a significant finding for neoplastic diseases, because the T7 vertebra was already fractured at the time of the initial examination. The patient had been receiving MTX for a long time, which led us to suspect MTX-LPD. Immediately after MTX was discontinued, thoracic spinal fixation from T5 to T9 was performed for the T7 compression fracture. Postoperatively, the patient's back pain resolved immediately, and she was able to perform activities of daily living independently. The tumour regression was observed on MRI two weeks after MTX withdrawal and the postoperative CT and MRI performed 30 months after MTX withdrawal showed complete union of the T7 vertebra and no evidence of tumour recurrence. Although LPD disappeared after MTX withdrawal, careful follow-up is necessary for at least two years because of the risk of tumour recurrence.

## Conclusions

Here, we describe a rare case of MTX-LPD at the T7 vertebra with a compression fracture. Although the vertebral fractures made it difficult to achieve a definitive diagnosis of MTX-LPD, MRI findings and local biopsy were the most helpful inspections in this case. When the fractured vertebra has a possibility of lymphoma or MTX-LPD and no evidence of cancer metastasis from pathological examination, the posterior spinal fixation and MTX withdrawal could be a first-line treatment. If a tumour regression and improvement of clinical symptoms are obtained after MTX withdrawal, a definitive diagnosis of MTX-LPD becomes available.
